# The impact of farming on prehistoric culinary practices throughout Northern Europe

**DOI:** 10.1073/pnas.2310138120

**Published:** 2023-10-16

**Authors:** Alexandre Lucquin, Harry K. Robson, Ester Oras, Jasmine Lundy, Giulia Moretti, Lara González Carretero, Joannes Dekker, Özge Demirci, Ekaterina Dolbunova, T. Rowan McLaughlin, Henny Piezonka, Helen M. Talbot, Kamil Adamczak, Agnieszka Czekaj-Zastawny, Daniel Groß, Witold Gumiński, Sönke Hartz, Jacek Kabaciński, Satu Koivisto, Trond Eilev Linge, Ann-Katrin Meyer, Teemu Mökkönen, Bente Philippsen, Gytis Piličiauskas, Vanda Visocka, Aivar Kriiska, Daan Raemaekers, John Meadows, Carl Heron, Oliver E. Craig

**Affiliations:** ^a^BioArCh, Department of Archaeology, University of York, York YO10 5DD, United Kingdom; ^b^Institute of History and Archaeology, Institute of Chemistry, University of Tartu, Tartu 50411, Estonia; ^c^Swedish Collegium for Advanced Study, Uppsala 752 38, Sweden; ^d^The British Museum, London WC1B 3DG, United Kingdom; ^e^Section for Geobiology, Globe Institute, University of Copenhagen, Copenhagen 1350, Denmark; ^f^Groningen Institute of Archaeology, University of Groningen, Groningen 9712, Netherlands; ^g^Department of Archaeology of Eastern Europe and Siberia, State Hermitage Museum, Saint Petersburg 190000, Russia; ^h^Institute of Prehistoric Archaeology, Department of History and Cultural Studies, Free University, Berlin 14195, Germany; ^i^Institute of Archaeology, Faculty of History, Nicolaus Copernicus University, Toruń 87-100, Poland; ^j^Centre for Archaeology of Hills and Uplands, Institute of Archaeology and Ethnology, Polish Academy of Sciences, Kraków 00-927, Poland; ^k^Museum Lolland-Falster, Nykøbing F. 4800, Denmark; ^l^Faculty of Archaeology, University of Warsaw, Warsaw 00-927, Poland; ^m^Stiftung Schleswig-Holsteinische Landesmuseen, Schloss Gottorf, Schleswig 24837, Germany; ^n^Department of Archaeology, University of Turku, Turku FI-20014, Finland; ^o^University Museum of Bergen, Section for Cultural Heritage Management, Bergen 5007, Norway; ^p^Institute of Prehistoric and Protohistoric Archaeology, University of Hamburg, Hamburg 20146, Germany; ^q^Cultural Environment Services, The Finnish Heritage Agency, Helsinki 913, Finland; ^r^NTNU University Museum, Norwegian University of Science and Technology, Trondheim NO-7491, Norway; ^s^Lithuanian Institute of History, Vilnius 01101, Lithuania; ^t^Department of History and Archaeology, Faculty of History and Philosophy, University of Latvia, Rīga 1050, Latvia; ^u^Department of Archaeology, Institute of History and Archaeology, University of Tartu, Tartu 50090, Estonia; ^v^Centre for Baltic and Scandinavian Archaeology, Schleswig 24837, Germany

**Keywords:** pottery, hunter-gatherers, early farmers, organic residue analysis, circum-Baltic

## Abstract

To investigate changes in culinary practices associated with the arrival of farming, we analysed the organic residues of over 1,000 pottery vessels from hunter-gatherer-fisher and early agricultural sites across Northern Europe from the Lower Rhine Basin to the Northeastern Baltic. Here, pottery was widely used by hunter-gatherer-fishers prior to the introduction of domesticated animals and plants. Overall, there was surprising continuity in the way that hunter-gatherer-fishers and farmers used pottery. Both aquatic products and wild plants remained prevalent, a pattern repeated consistently across the study area. We argue that the rapid adaptation of farming communities to exploit coastal and lagoonal resources facilitated their northerly expansion, and in some cases, hunting, gathering, and fishing became the most dominant subsistence strategy. Nevertheless, dairy products frequently appear in pottery associated with the earliest farming groups often mixed with wild plants and fish. Interestingly, we also find compelling evidence of dairy products in hunter-gatherer-fisher Ertebølle pottery, which predates the arrival of domesticated animals. We propose that Ertebølle hunter-gatherer-fishers frequently acquired dairy products through exchange with adjacent farming communities prior to the transition. The continuity observed in pottery use across the transition to farming contrasts with the analysis of human remains which shows substantial demographic change through ancient DNA and, in some cases, a reduction in marine consumption through stable isotope analysis. We postulate that farmers acquired the knowledge and skills they needed to succeed from local hunter-gatherer-fishers but without substantial admixture.

## Significance

How prehistoric farming became established in Northern Europe, a region that supported dense populations of hunter-gatherer-fishers, has concerned archaeologists for over a century. Through analysis of the organic residues recovered from over 1,000 vessels dating across the transition to farming, we found unexpected consistency in the use of aquatic foods at odds with prevailing narrative of large-scale demographic and economic change. We argue that the ability of farming groups to adapt to their environment by learning hunter-gatherer-fisher practices, combined with dairying, was key to their northerly expansion. We also provide evidence of dairy use by hunter-gatherers which we attribute to long-distance exchange with farmers, implying a much greater degree of interaction and cooperation than previously described.

Farming transformed societies globally, leading ultimately to the creation of large sedentary populations, more pronounced social inequality, and profound impacts on human health. Understanding the transition to food production, be it a gradual acceptance or sudden imposition, and the impact on our hunter-gatherer forebears continues to be one of the great challenges in prehistoric archaeology, despite over a century of study (1, 2). Demographic expansion of farmers, with domesticated plants and animals, into sparsely occupied, near pristine territories remains an alluring and easily grasped view of this process. For many regions in Europe, this view appears to have been bolstered by the analysis of ancient human genomes, which show little admixture between farmers and indigenous hunter-gatherers (3–5). Nevertheless, farmers inevitably met substantial numbers of foragers, with different ancestries, foodways, and notions of the world. Across Northern Europe and the circum-Baltic during the mid-Holocene, such encounters must have happened multiple times. This was a period when hunter-gatherer-fishers flourished in semisedentary, surplus-producing societies, on the borders of forests, coasts, and rivers rich in wild, particularly aquatic, resources (1). Here, it is harder to understand how, why, and the extent to which farming ultimately took hold.

At its heart, the transition to farming relates to a fundamental change in the relationship between humans and food. Consequently, studies of faunal and botanical assemblages and stable isotope analyses of human remains, to track changes in diet, have provided some of the most useful datasets for understanding the transition (6–8). Although immensely useful for studying the productive economy, these studies often reveal little about how foodstuffs were processed and consumed. The latter are cultural elements related to the treatment of foodstuffs rather than subsistence. In many cases, organic remains are poorly preserved leading to a partial record with which to draw conclusions. This problem is exacerbated at very early farming sites where domesticated resources may have made only a minor contribution to the overall economy or diet. Here, we take a different approach involving the comparative analysis of the use of ceramic containers before and after the arrival of farming.

Unlike animal and plant remains, prehistoric ceramics are the most abundant archaeological findings preserved in almost all depositional environments. In contrast to much of Europe, hunter-gatherer-fishers independently produced ceramic vessels throughout the circum-Baltic and parts of Northwestern Europe before farming (9), providing an opportunity to directly compare pottery use across the transition. Recovery of traces of organic remains from forager pottery in this region has already provided detailed culinary insights and patterns about the types of prepared foods (e.g., aquatic, wild ruminant, foraged plant), regardless of environmental setting (10, 11). Across this region, the arrival of domesticated plants and animals was often concomitant with new, exogenous pottery types and other forms of material culture. But what impact did these changes have on the way pottery was used and more broadly on foodways? An obvious answer is that the function of new pottery types was focused on newly acquired domesticated resources, such as dairy products and cereals, marking a fundamental technological shift regardless of the overall scale of subsistence change.

**Fig. 1.**
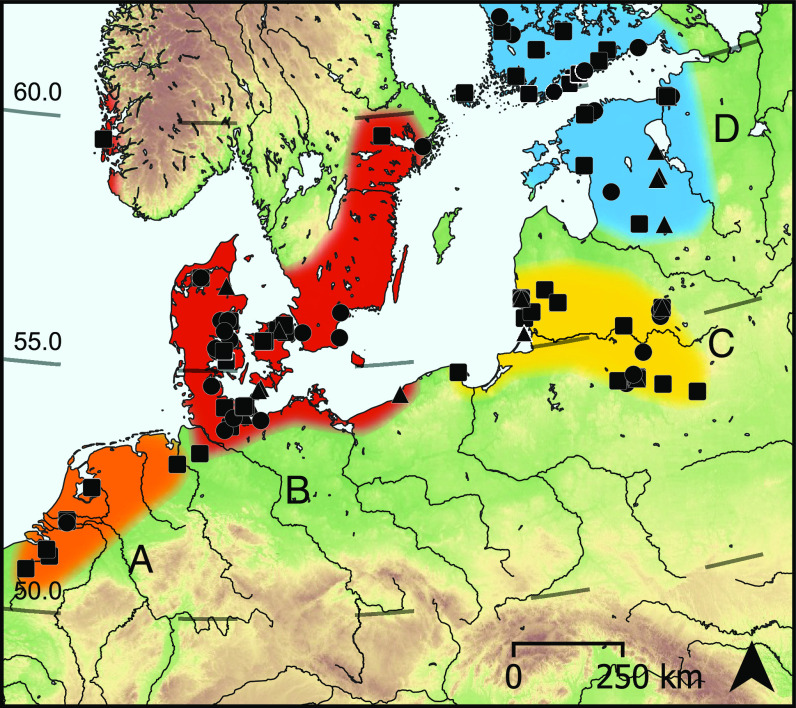
Map of the study area showing the diachronic set of sites, dated between the 5th and 3rd millennia cal BCE, and the regional divisions (*A*–*D*) used in this study. Sampled archaeological sites with typical hunter-gatherer-fisher pottery (circle), early farmer pottery (square), or both (triangle) are shown. Individual sites are listed in *SI Appendix,* Fig. S1 and Dataset S1.

Small-scale regional studies have already attempted to investigate this question, but have produced conflicting views of the degree of continuity or change (12–15). Here, we expand this approach to cover the entire region from the Lower Rhine basin in Northwestern Europe to the Baltic countries in the north-east (Fig. 1), encompassing 132 archaeological sites (*SI Appendix,* Fig. S1). The arrival of farming is defined by the earliest presence of domesticated animals or crops in each region and ranges from ca. 4300 cal BCE in the west to ca. 2500 cal BCE in the east, and with the exception of the Swifterbant (SWB) cultural group, is generally associated with the introduction of new “Neolithic” pottery types. The study area is divided into the following regions based on broad chronological and cultural attributes (Fig. 1):

- a. Lower Rhine basin (Fig. 1*A*), where domesticated animals and cultivated cereals appear within the SWB cultural groups at around 4300 BCE (16) prior to the appearance of exogenous Neolithic material culture [Funnel Beaker (TRB) and Michelsberg] in the 4th millennium cal BCE.
- b. Central and Western Baltic (Fig. 1*B*), where Ertebølle (EBK) and other Mesolithic wares were replaced by Neolithic TRB pottery ca. 4000 cal BCE. These also include TRB pottery from Sweden dating to the 4th millennium cal BCE and a Middle Neolithic local pottery tradition from western Norway dating to the 4th-3rd millennium cal BCE.
- c. Southeastern Baltic, where Porous ware pottery was replaced by Rzucewo (RW) and Globular Amphora ware (GAC) toward the end of the 4th millennium cal BCE and later by Corded Ware (CWC) in the early 3rd millennium cal BCE (14).
- d. Northern and Eastern Baltic, where CWC pottery was introduced in the early 3rd millennium cal BCE succeeding Comb Ware (CBW) ceramics (17); however, these and other locally defined forms of hunter-gatherer-fisher pottery likely persisted in parallel throughout the 3rd millennium cal BCE.

We compiled a dataset (n = 598) of molecular and isotopic measurements of lipids extracted from vessels typically associated with the earliest agricultural communities (SWB, Michelsberg, TRB, RW, GAC, and CWC). While not all the vessels were from sites with direct evidence of domesticated plants and animals, they are chronologically and culturally related with evidence of farming in their respective regions. These data were then compared with a dataset (n = 555) of hunter-gatherer-fisher potsherds from the same geographic area. The total dataset consisted of published data from 476 samples, reanalysed molecular and isotopic lipid data from 481 samples, and newly generated data obtained by lipid analysis from a further 196 samples, corresponding to a total of 1,080 vessels (Dataset S1). Furthermore, we compiled the results of published (n = 598) and new (n = 186) measurements of bulk carbon and nitrogen isotopes of charred deposits (Dataset S1).

## Results and Discussion

### The Establishment of Dairying in Northern Europe.

Dairy products, including raw milk and its fermented products (e.g., yoghurts, cheese, and butter) have a high fat content and are readily absorbed in pottery vessels. Their lipids can be distinguished isotopically from other food sources due to a large difference (Δ^13^C) between the δ^13^C values of the principal C_16:0_ and C_18:0_ fatty acids (18). Previous work has shown that early farmers from across Europe regularly used pottery containers to process milk despite low levels of lactase persistence (19–21). Dairy has been detected in pottery from Early Neolithic farming groups of the Northern European plain belonging to the Linear Pottery culture (LBK) during the 6th millennium cal BCE and among successive Neolithic groups (21). The interaction of these farmers with adjacent hunter-gatherer-fishers living along the Baltic and in the Lower Rhine Basin has long been debated (22).

**Fig. 2.**
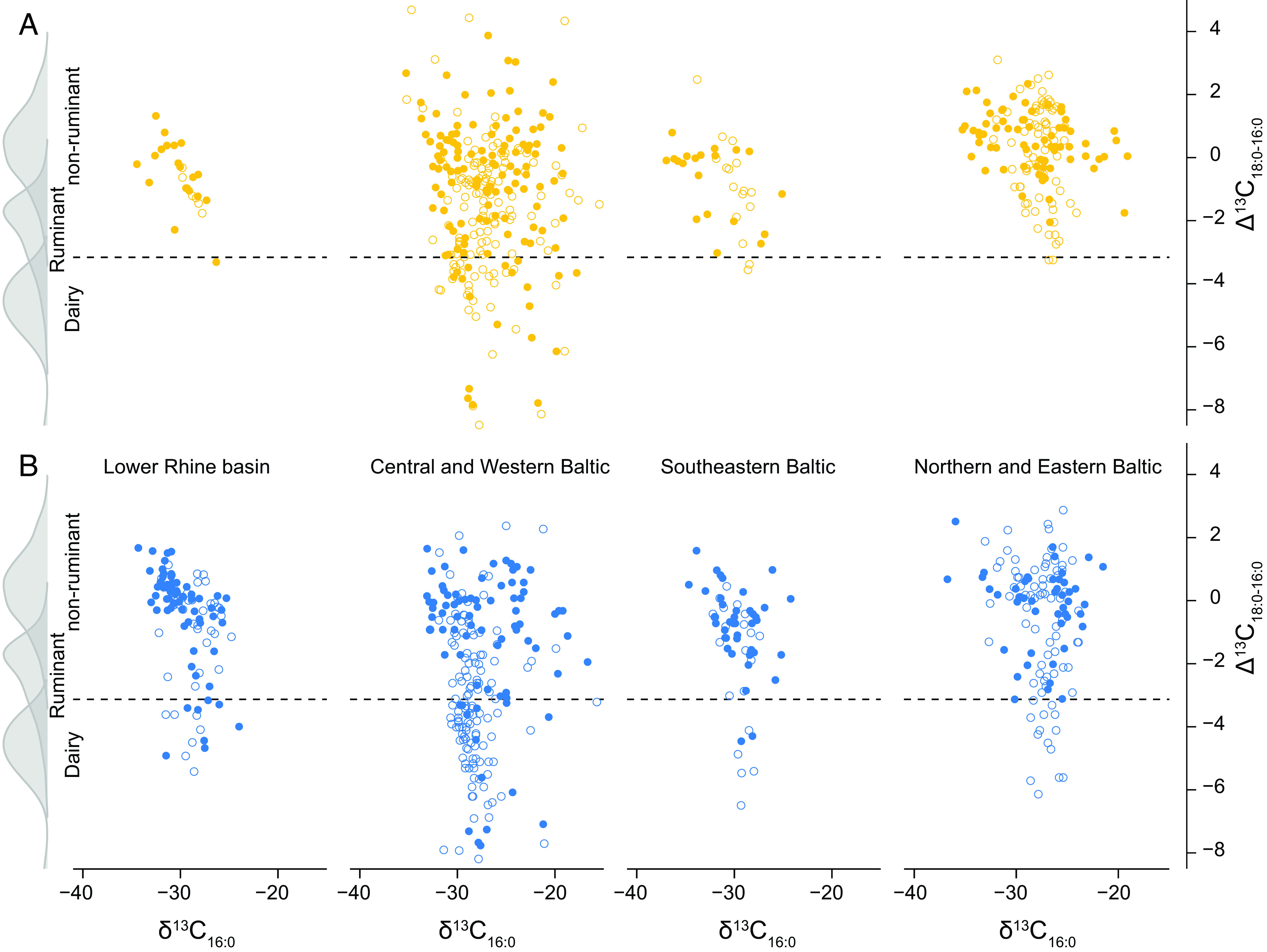
Scatter plots of δ^13^C_16:0_ against Δ^13^C values (δ^13^C_18:0_ − δ^13^C_16:0_) from pottery typically associated with hunter-gatherer-fishers (*A*) and early farmers (*B*) from the Lower Rhine Basin (SWB and Michelsberg), Central and Western Baltic (EBK, Mesolithic ware, and TRB), Southeastern Baltic (Subneolithic, Porous, RW, GAC, and CWC), and Northern and Eastern Baltic (CBW, Porous, JW, GAC, and CWC). The data are compared with modern reference ranges expressed as densities. The upper Δ^13^C limit for dairy products (−3.1‰) after (21) is shown. The samples containing aquatic-derived lipids are expressed as filled circles (Dataset S1).

**Fig. 3.**
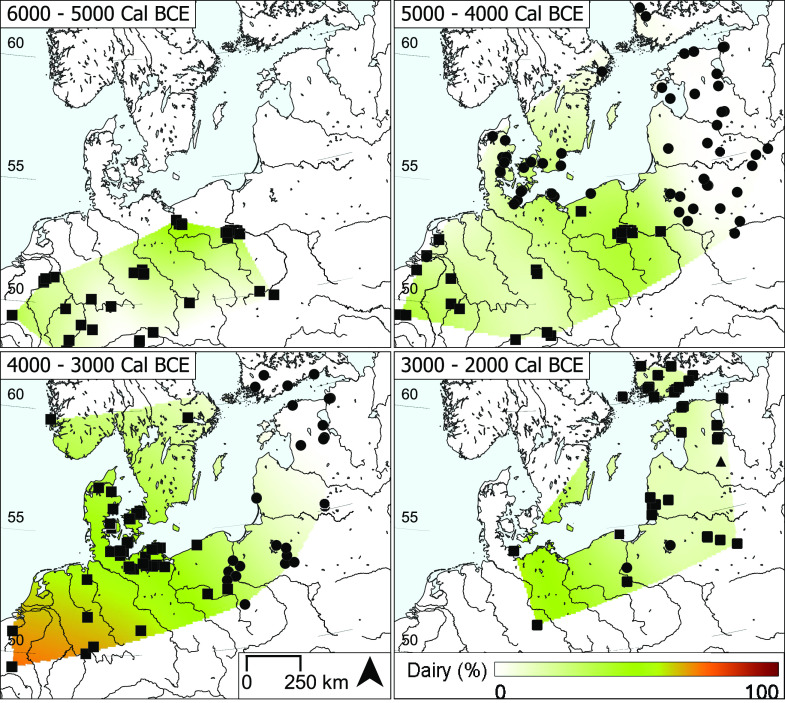
Spatial interpolation of the frequency of dairy fat residues (Δ^13^C <−3.1‰) across Northern Europe shown in millennia time slices. Sites with typical hunter-gatherer-fisher pottery type (circle), early farmer pottery type (square), or both (triangle) are shown. Errors associated with the interpolation are shown in *SI Appendix*.

We detected dairy products in early farmer pottery from each of the four regions investigated (Fig. 2*B*), using an upper Δ^13^C limit of <−3.1‰ (21). Dairy was more prevalent in TRB pottery from the Western and Central Baltic than early farmer pottery from the other investigated regions (Fig. 2*B*). When the data are compared with the larger dataset of inland LBK, post-LBK and TRB groups (Fig. 3), the prevalence of dairy did not diminish with increasing proximity to the coast, suggesting that there was little to impede this practice (*SI Appendix, Geographical Attributes*). The frequency of dairy in TRB (46%, n = 454) and Michelsberg pottery (69%, n = 97) is much higher than in earlier LBK groups throughout the region and beyond (26%, n = 1,251; Dataset S2). In the Eastern Baltic, dairy is equally prevalent in CWC ceramics (19%, n = 93) as in other Neolithic pottery (RW, GAC, 18%, n = 68). Here, it seems likely that dairy was introduced by the arrival of pastoralists with Steppe or Central European ancestry (23, 24).

In the Central and Western Baltic, the available human DNA data supports a clear demographic transition with the arrival of farming (3, 25). Surprisingly, however, a significant proportion (22%, n = 214) of hunter-gatherer-fisher EBK pots from this area plot within the dairy range (Fig. 2*A*), both from this study and elsewhere (21). Dairy products were identifiable in forager pottery even when more stringent criteria are applied which account for lower Δ^13^C values observed in wild ruminant fats (26). As there is no compelling evidence for domesticated animals, except dogs, at EBK sites (27), regular exchange of dairy products between post-LBK farming communities and EBK hunter-gatherer-fishers is postulated to explain this finding. Exchange of durable raw materials and finished goods between these groups throughout this region is well attested (28–32), but the movement of perishable items, such as dairy products, even if they were heavily fermented must have required rapid distribution across the agricultural “frontier”, implying a deeper level of cooperation. If so, hunted and foraged perishable goods, such as furs and marine mammal oil, could have been exchanged with farmers as previously suggested (33), although such connections are difficult to demonstrate. In such a scenario, inflationary demand for domesticated resources has been argued as a mechanism for undermining the forager subsistence system, leading to the establishment of food production (33, 34).

Raw Δ^13^C values are also heavily influenced by mixing of products with different relative amounts of fatty acids. Mixing of products was more prevalent in EBK (Fig. 2*B*) pots compared to the TRB (Fig. 2*A*), due to the broader range of observed δ^13^C_16:0_ values. In some cases, mixtures of dairy fats and aquatic oils provide a convincing interpretation and there is little evidence that dedicated EBK vessels were reserved for dairy. A similar pattern is observed in the SWB sample (Fig. 1*A*). Conversely, both early farmer TRB and CWC pots associated with dairy products have a narrower range of δ^13^C_16:0_ values and fewer or no aquatic lipid biomarkers, suggesting some separation from other inputs (Fig. 2). These data point to an interesting conceptual change in how dairy foods were incorporated into wider culinary practices, perhaps related to a proliferation of vessel forms (35).

### Continuation of Aquatic Resource Use beyond the Transition to Farming.

The degree to which aquatic resources were exploited beyond the arrival of agriculture has attracted substantial debate, drawing largely upon stable isotope analysis of human remains (36). In many areas of Northern Europe, there is clear evidence of aquatic resource consumption beyond the arrival of domesticated plants and animals (8, 37, 38). However, a more abrupt dietary change from aquatic to terrestrial foods with the transition to farming has been demonstrated through stable isotope analysis of human bone in both Denmark (6) and the Eastern Baltic (14). In contrast, we found that early farmer pottery from across the study area was frequently used for processing aquatic foods.

Aquatic lipid signatures, comprising isoprenoid fatty acids (phytanic, pristanic, or 4,8,12-trimethyltridecanoic acid) and *ω*-(*o*-alkylphenyl)alkanoic acids (APAAs) with C_18_ and C_20_ carbon atoms (39–41), were found in 41% (*234/573*) of the samples with preserved lipids dating after the appearance of farming compared with 42% (*222/534*) of the hunter-gatherer-fisher vessels. Furthermore, about 28% of the samples (*102/360*, pre-farming; *85/307*, farming) produced a ratio (SRR%) of the two naturally occurring diastereomers of phytanic acid above 75.5% that can be assigned to aquatic animals, using a conservative limit (11). At Dąbki, in northern Poland, analysis of locally made Mesolithic wares and later Neolithic TRB ceramics show no perceptible change in use. Here, aquatic biomarkers are prevalent in both periods and carbon isotope values of fatty acids span the range expected for local freshwater resources (ca. −37 to −25‰). Overall, distance from the coast or river does not influence the likelihood that aquatic biomarkers are present at either hunter-gatherer-fisher or farming sites (*SI Appendix, Geographical Attributes*), suggesting that these foods were widely consumed. Nevertheless, we observe a significant decrease in the variance in the δ^13^C value of the C_16:0_ fatty acid at the onset of farming globally (Levene’s test, F = 26.1, *P* < 0.005, variances 13.8 and 8.1), which may reflect a relatively greater input of terrestrial animal products at this juncture, with less varied δ^13^C values compared to aquatic resources.

The data from early farmer pottery across the study area contrast sharply with other European Neolithic regions, where aquatic products are virtually absent in pottery. Importantly, this includes data from studies that have used similarly sensitive approaches for their detection. For example, in the United Kingdom, France, and the Iberian Peninsula, there is no evidence of aquatic foods during the Early Neolithic, even in pottery from coastal sites where fishing was at least a minor subsistence activity (19). One of the only exceptions noted so far is the presence of substantial amounts of aquatic products in Early Neolithic Starčevo–Körös–Criş pottery from the Danube gorges in Southeastern Europe (42). As with the circum-Baltic, these early farming groups were culturally influenced by semisedentary hunter-gatherer-fishers living along the gorges at high density (43).

### Evaluating the Contribution of Plant Foods through Lipid Biomarkers, EA-IRMS (Elemental Analysis-Isotope Ratio Mass Spectrometry), and Microscopic Investigation.

A shift to cereal processing in pottery associated with the implementation of agricultural regimes is difficult to demonstrate using lipid residue analysis, due to issues with overprinting and poor preservation of cereal biomarkers, especially at dryland sites (44). Lipids derived from plant oils and waxes, such as long-chain alkanes with odd carbon numbers, even-numbered long-chain fatty acids, amyrin and its derivatives as well as phytosterols (Dataset S1), were identified in a range of samples, but these occur in both wild and domesticated plants. A complementary approach involves considering the nitrogen isotope values (δ^15^N) and atomic carbon to nitrogen (C:N) ratios of the charred deposits (“foodcrusts”) observed adhering to the insides of many of the vessels. Nitrogen derived from plants rather than terrestrial animals and aquatic proteins can be crudely identified through lower δ^15^N values and higher C:N ratios (45). Determination of bulk foodcrust compositions may better reflect plant processing as their major constituents, i.e., starches, sugars, fibre, and proteins, are less amenable to biomolecular analysis, especially if they have been thermally degraded through the charring process.

**Fig. 4.**
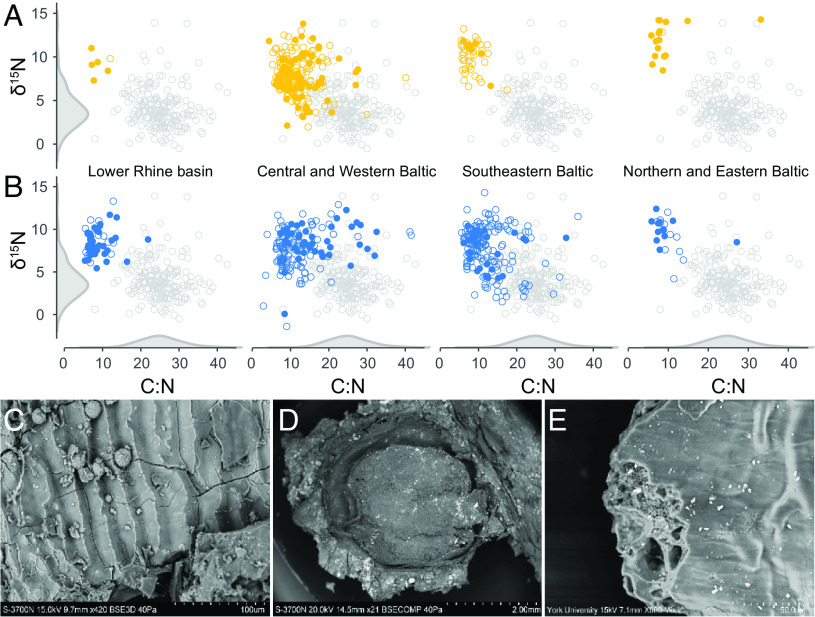
Nitrogen stable isotope values of charred surface deposits from prehistoric hunter-gatherer-fisher (*A*—yellow) and early farmer (*B*—blue) pottery from the Lower Rhine Basin (SWB and Michelsberg), Central and Western Baltic (EBK, Mesolithic ware, and TRB), Southeastern Baltic (Subneolithic, Porous, RW, GAC, and CWC), and Northern and Eastern Baltic (CBW, Porous, JW, GAC, and CWC). The δ^15^N values for each region are plotted against the atomic C:N ratios. Filled symbols indicate the identification of aquatic biomarkers in the charred deposit. The reference ranges for TRB charred cereals are shown on the axes (Dataset S1). SEM micrographs show examples of wild and domesticated plant and animal resources identified in the charred deposits: *C*—cyprinid fish scales (Dąbki), *D*—berries of *Viburnum opulus* (Dąbki), and *E*—emmer wheat pericarp (Syltholm II).

From the analysis of foodcrusts adhering to 308 hunter-gatherer-fisher vessels and 476 vessels associated with early farming, it is evident that ≈90% of samples have δ^15^N values and atomic C:N ratios outside the range of cereals previously measured from the study area (Fig. 4 and Dataset S1), indicating a significant contribution from higher trophic-level, more proteinaceous terrestrial and aquatic animal resources (45). This interpretation is supported by the presence of aquatic-derived lipids (Fig. 4) and the presence of dairy in many of the vessels. A small number of pots associated with early farming from the Northern and Eastern Baltic (Fig. 4*I*) have atomic C:N ratios and δ^15^N values consistent with cereals, but wild plants are equally plausible. In other cases, mixtures of plant and animal resources may have partly contributed to the foodcrust through multiple or concurrent charring events that are hard to quantify using this approach. Overall, there is no significant decline in δ^15^N (Mann–Whitney *U* test, W = 57,003, *P* = 0.05) or concomitant increase in the atomic C:N ratios (W = 67,696, *P* = 0.15), as would be expected if plant products made a more prominent contribution following the arrival of agriculture.

To investigate this further, we carried out high-resolution microscopic analysis of 55 foodcrusts adhering to pottery vessels across the study area (Dataset S1). The results show that pottery vessels associated with foragers (n = 33) and early farmers (n = 22) were frequently used for processing wild plants (e.g., berries, grasses, forbs, and roots/tubers), but in combination with aquatic resources (e.g., fish), and these foodcrusts are therefore unlikely to have similar elemental and isotopic characteristics to charred plant remains. Three TRB foodcrust samples from the site of Syltholm II in Southern Denmark yielded remains of cereal grains (emmer wheat and barley), but these are always found in mixtures with either wild plants or fish and were unlikely to have been the main food component. Similarly, cereals (emmer wheat and barley) mixed with wild resources have been microscopically identified in Late SWB foodcrusts from the Lower Rhine basin (46). No cereals have been identified in CWC or GAC pottery to date.

### Pottery Use and the Transition to Farming in Northern Europe.

The role of pottery in late forager and early farming societies in Northern Europe does not conveniently fit with existing narratives regarding the transition to farming, which often invoke the extremes of continuity or change, migrationism, or indigenism. As with many other regions of Europe, the identification of milk fat in pottery at the earliest sites with domesticates underlines the importance of dairying for the dispersal of the farming Neolithic, especially to northern latitudes (19). However, it seems likely that Mesolithic foragers in Denmark and Northern Germany acquired dairy products through exchange with adjacent Neolithic farmers prior to the arrival of domesticated animals or genetic admixture with farmers. Whether contact with farmers had a destabilising effect on Mesolithic hunter-gatherer-fishers is debatable (34, 47), as we cannot assume that exchanged items were ascribed higher prestige than locally produced goods. The fact that dairy products became enmeshed in late forager foodways and were moved, most conceivably as ferments, shows that the boundaries between these cultural groups immediately prior to the transition were much more diffuse than previously thought.

A “Negotiation phase” which emphasises the active role of hunter-gatherer-fishers in facilitating the transition to farming has been proposed in Southern Scandinavia (27). However, this model predicts genetic exchange between foragers and farmers, which is not easily supported by current genomic data available for Denmark, showing almost no evidence of individuals with hunter-gatherer ancestry in subsequent generations beyond the arrival of farming (3). Critically, without some degree of heterogamy, it is questionable whether foragers could ever adopt farming (48). In the Eastern Baltic the genetic data are currently much sparser. Here, the earliest farmers belonging to the CWC had predominantly Steppe and, to a lesser degree, Anatolian ancestry, also pointing to profound demographic change at this juncture with limited genetic admixture during the subsequent centuries of coexistence between CBW and CWC populations (3, 23, 49).

Here, we propose an alternative hypothesis to explain continuity in the use of pottery despite significant genetic and cultural change, i.e., that Neolithic farmers (TRB, GAC, and CWC) adapted their practices to accommodate the rich littoral, estuarine, and lagoonal resources afforded by the northerly environments they expanded into. The continued exploitation of aquatic resources in this region is less easily explained by the existence of isolated pockets of hunter-gatherer-fishers, producing TRB pottery, as in many cases dairy foods and, in some cases, domesticated animal bones are present in the same assemblages. Indeed, there is evidence that both wild and domesticated produce were occasionally prepared together in the same vessels, such as at Syltholm II in southern Denmark (Dataset S1).

Expansion of farming groups from the Northern European plain to coastal and estuarine environments must have presented new opportunities for fishing and shellfish gathering. It is plausible that farmers learned how to most effectively exploit these rich aquatic ecotones, from indigenous hunter-gatherer-fishers either through direct observation or participation, including the use of pots for processing local wild resources. While this scenario is a reasonable mechanism to explain our dataset, it relies on a degree of asymmetry, i.e., that hunter-gatherers were unable to independently learn farming whereas farmers were easily able to adapt hunting, gathering, and fishing strategies.

This scenario cannot be applied to regions where there are no ancient genomic data, including the Lower Rhine basin. In this region, the adoption of elements of farming by foragers through intermarriage with farming communities remains plausible. In contrast, in the Northeastern Baltic, hunter-gatherer-fishers and farmers likely coexisted during the 3rd millennium BCE (17), but dairy products are only found associated with the CWC, along with aquatic foodstuffs, suggesting some distinction in pottery use between these groups. Here, as in other parts of the Baltic, our data do not necessarily contradict a broad shift from aquatic to terrestrial diets highlighted in the human stable isotope data (6, 14). Human bone collagen isotopes provide semiquantitative dietary estimations usually of relatively few individuals, often buried selectively, whereas pottery residues reflect culinary practices incurred by households and communities but provide no insight into aceramic food preparation nor a quantitative measure of overall food supply. We can conclude, however, that dietary change was not absolute; some culinary traditions were clearly retained despite the commencement of food production and changes in pottery manufacturing techniques. We note that cultural traits involving food may have been inherently more robust in evolutionary terms than other traditions, as they were intrinsically adapted to specific environmental settings.

## Materials and Methods

### Organic Residue Analysis.

Lipids from pottery vessel sherds and charred deposits on vessel surfaces were extracted and methylated in one step with acidified methanol (H_2_SO_4_:MeOH, 1:5) following detailed published methods (10). Briefly, methanol was added to homogenised carbonised residues (10 to 20 mg) or drilled/crushed ceramic powders (0.5 to 1.0 g), sonicated for 15 min, and acidified with concentrated sulphuric acid, and then, the acidified suspension was heated for 4 h at 70 °C. The lipids were extracted by phase separation with *n*-hexane (3 × 2 mL) and concentrated under a gentle flow of nitrogen. The general screening of the extract was realised by gas chromatography–mass spectrometry (GC-MS) in total ion current mode. A dedicated selected ion monitoring mode was used to detect specific markers of aquatic resources (isoprenoids and APAAs). Carbon isotope values of the main fatty acids methyl esters (C_16:0_ and C_18:0_) were acquired by GC–combustion–isotope ratio MS. Interior or exterior charred residues were also analysed by EA-IRMS, using previously reported protocols (14).

### Microscopic Analysis of Foodcrusts.

Sherds with adhered foodcrusts were selected for high-resolution microscopic analysis. Initial observation was carried out using a low-powered Leica MZ APO binocular microscope at magnifications of between 8× to 50×. Images of the foodcrusts’ appearance and microstructure were created using a VHX-5000 Keyence digital microscope at magnifications from 20× to 200×. Subsequently, foodcrusts were further studied under a scanning electron microscope (SEM) using a Hitachi TM4000 at the University of York. Plant and animal inclusions and voids were estimated, quantified, measured, and categorised after González Carretero et al. (50).

### Statistical and GIS.

To generate spatial estimates of the frequency of sample with Δ^13^C <−3.1‰, we employed the AverageR models available as R-based Open Access apps (https://www.isomemoapp.com/) developed within the Pandora & IsoMemo initiatives. Mapping was undertaken with QGIS (version 3.28.2-Firenze) using Natural Earth. Point pattern and proximity analyses were performed using GRASS 7.4.4 (grass.osgeo.org). All statistical tests were performed using *R* studio (version 2022.07.2 Build 576).

## Supplementary Material

Appendix 01 (PDF)

Dataset S01 (XLSX)

Dataset S02 (XLSX)

## Data Availability

All study data are included in the article and/or supporting information.
