# Supplementary material for: The impact of farming on prehistoric culinary practices throughout Northern Europe
Source: Proc Natl Acad Sci U S A. 2023 Oct 16;120(43):e2310138120. doi: 10.1073/pnas.2310138120 (PMC10614617; doi:10.1073/pnas.2310138120)
Supplement: Supplementary file 1 — Appendix 01 (PDF) [file pnas.2310138120.sapp.pdf]

PNAS

Supporting Information for

The impact of farming on prehistoric culinary practices throughout Northern Europe

Alexandre Lucquin[1]*, Harry K. Robson[1], Ester Oras[2,3], Jasmine Lundy[1], Giulia Moretti[4], Lara González Carretero[1], Joannes Dekker[1,5], Özge Demirci[1,6], Ekaterina Dolbunova[4,7], T. Rowan McLaughlin[4], Henny Piezonka[8], Helen M. Talbot[1], Kamil Adamczak[9], Agnieszka Czekaj-Zastawny[10], Daniel Groß[11], Witold Gumiński[12], Sönke Hartz[13], Jacek Kabaciński[10], Satu Koivisto[14], Trond Eilev Linge[15], Ann-Katrin Meyer[16], Teemu Mökkönen[17], Bente Philippsen[18], Gytis Piličiauskas[19], Vanda Visocka[20], Aivar Kriiska[21], Daan Raemaekers[6], John Meadows[22], Carl Heron[4] and Oliver E. Craig[1]

[1]BioArCh, Department of Archaeology, University of York, York, UK
[2]Institute of History and Archaeology, Institute of Chemistry, University of Tartu, Tartu, Estonia
[3]Swedish Collegium for Advanced Study (SCAS), Uppsala, Sweden
[4]The British Museum, London, UK
[5]Section for Geobiology, Globe Institute, University of Copenhagen, Copenhagen, Denmark
[6]Groningen Institute of Archaeology, University of Groningen, Groningen, Netherlands
[7]State Hermitage Museum, Saint Petersburg, Russia
[8]Institute of Prehistoric Archaeology, Free University, Berlin, Germany
[9]Institute of Archaeology, Nicolaus Copernicus University, Toruń, Poland
[10]Institute of Archaeology and Ethnology, Polish Academy of Sciences, Poland
[11]Museum Lolland-Falster, Nykøbing F., Denmark
[12]Faculty of Archaeology, University of Warsaw, Warsaw, Poland
[13]Stiftung Schleswig-Holsteinische Landesmuseen, Schloss Gottorf, Schleswig, Germany
[14]Department of Archaeology, University of Turku, Finland
[15]University Museum of Bergen, Section for Cultural Heritage Management, Bergen, Norway
[16]University of Hamburg, Hamburg, Germany
[17]The Finnish Heritage Agency, Helsinki, Finland
[18]Norwegian University of Science and Technology, Trondheim, Norway
[19]Lithuanian Institute of History, Vilnius, Lithuania
[20]Faculty of History and Archaeology, University of Latvia, Rīga, Latvia
[21]Institute of History and Archaeology, University of Tartu, Tartu, Estonia
[22]Centre for Baltic and Scandinavian Archaeology, Schleswig, Germany

These authors contributed equally: Alexandre Lucquin, Harry K. Robson

*Corresponding author: Alexandre Lucquin (alexandre.lucquin@york.ac.uk)

This PDF file includes:

- Supporting text
- Figures S1 to S3
- Tables S1
- Legends for Datasets S1 to S2
- SI References

Other supporting materials for this manuscript include the following:

- Datasets S1 to S2

Supporting Information Text

Generating the spatio-temporal distribution of prehistoric dairying across Northern Europe

To generate spatial estimates of the frequency of dairying, we employed the AverageR model available as R-based Open Access apps (https://www.isomemoapp.com/) developed within the Pandora & IsoMemo initiatives (Data search and Spatiotemporal modeling version 23.4.1.10).

AverageR is a generalized additive mixed model that uses a thin plate regression spline (1, 2). The dependent variable used was the percentage of dairy samples and was established by site/millenium according to the number of samples with $\Delta^{13}C$ <-3.1‰ against the total number of samples analyzed (Dataset S2). Four local average models were generated according to the following millennium time slices: 6th, 5th, 4th and 3rd millennia cal BCE.

Geographical attributes

To determine whether aquatic biomarkers were more prevalent on coastal sites or near rivers, the European Commission's Catchment Characterisation Model (CCM2) database (data.europa.eu/89h/fe1878e8-7541-4c66-8453-afdae7469221), was used to find the minimum distance between each site and the nearest river or tributary using the v.distance tool in GRASS GIS. This was also used to calculate the geodesic distance to the modern-day coast using the 1:10m global coastline map from Natural Earth (www.naturalearthdata.com). However, neither distance to coast nor distance to river can predict the presence or absence of aquatic biomarkers (Figure S3). The same is true for dairy biomarkers (Figure S3), both in the data reported in this paper and also the prehistoric dairying phenomenon in Central and Western Europe studied by Evershed et al. (3). Finally, in contrast to sites with hunter-gatherer pottery in the forest-steppe area of Eastern Europe (4), the sites in this study are not preferentially located close to navigable rivers when compared to a random distribution of points in the landscape (Figure S3). Whether this represents a deliberate change to settlement systems or is simply reflective of differences in catchment drainage and hypsometry requires further study.

Isotopic values of Early Neolithic Funnel Beaker culture charred cereals

Previous studies have found that TRB charred cereals from the western Baltic have a mean $\delta^{15}N$ value of 4.0‰ (±2.3‰ 1s.d., *n*=205) and an atomic C:N ratio of 25.2 (±5.4 1s.d., *n*=205) (5–9).

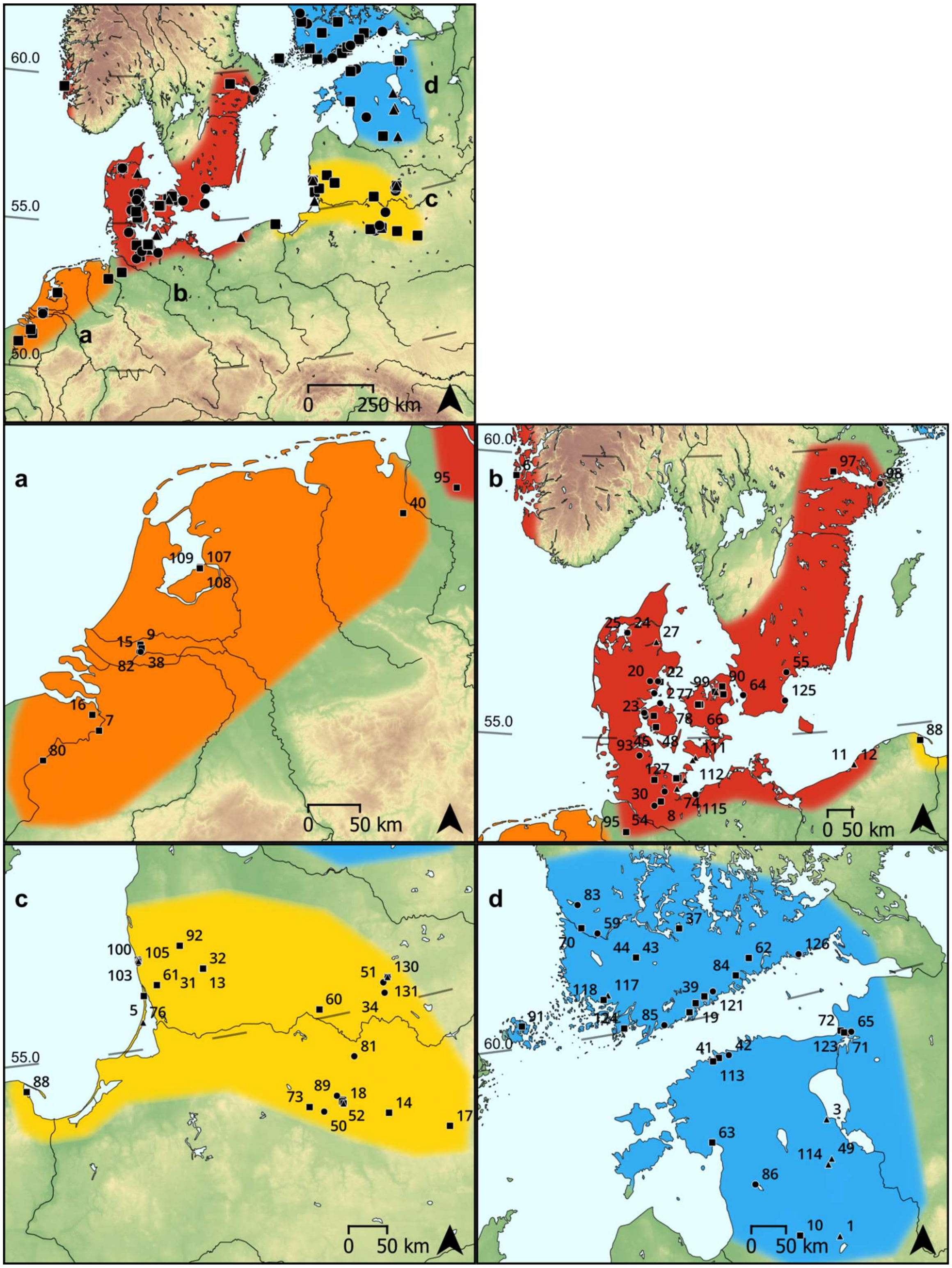

Fig. S1. Map of the study area (physical) showing the regional divisions (a-e) used in this study. Archaeological sites with typical hunter-gatherer pottery (circle), early farmer pottery (square) or both (triangle) are shown. The numbers are reported in Table S1.

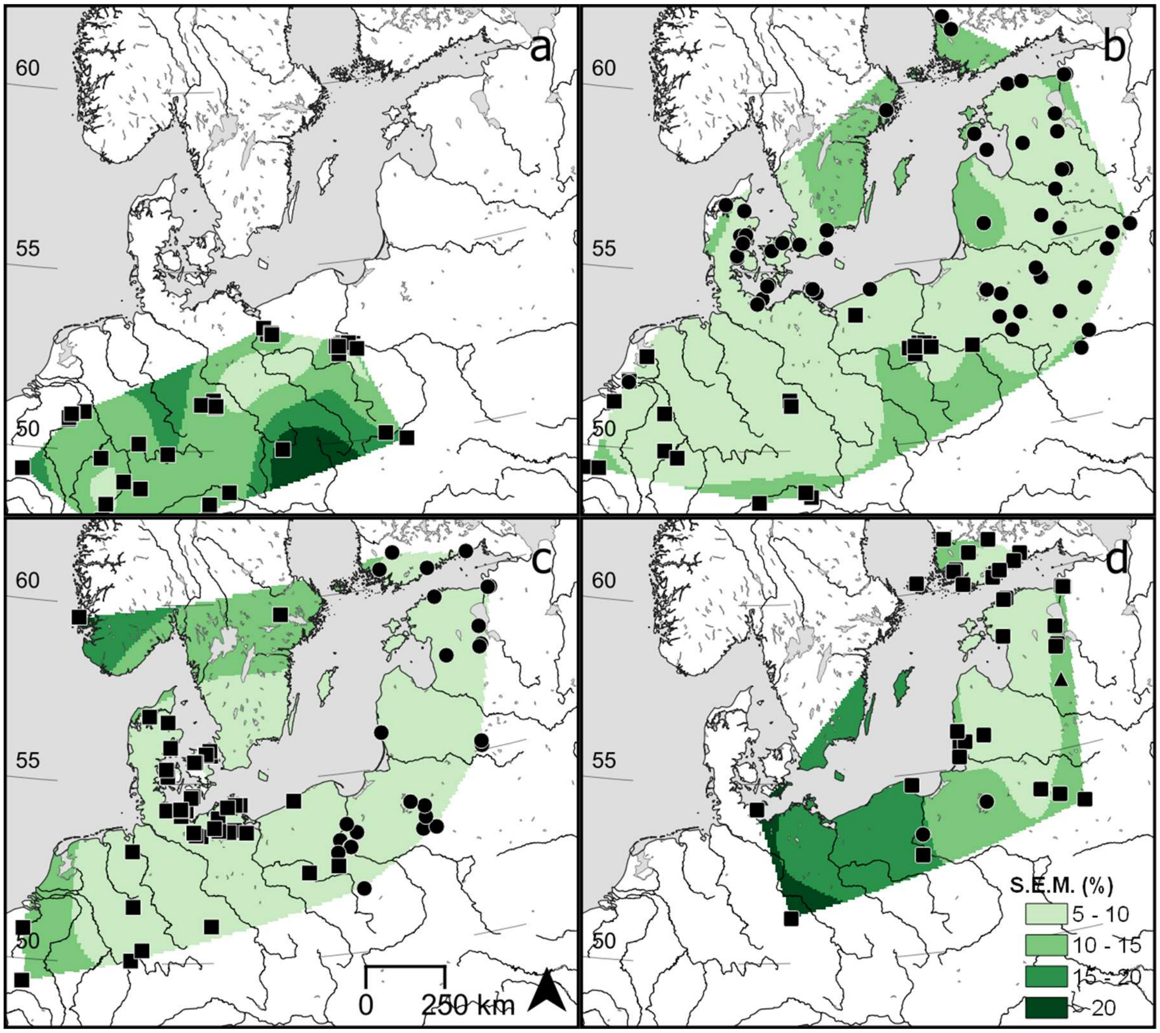

Fig. S2. Standard error of the mean (1σ) of the spatio-temporal distribution of prehistoric dairying across Northern Europe during the: (a) 6th, (b) 5th; (c) 4th; and (d) 3rd millennia cal BCE. Sites with (squares) and without (circles) evidence of domesticated animals is shown.

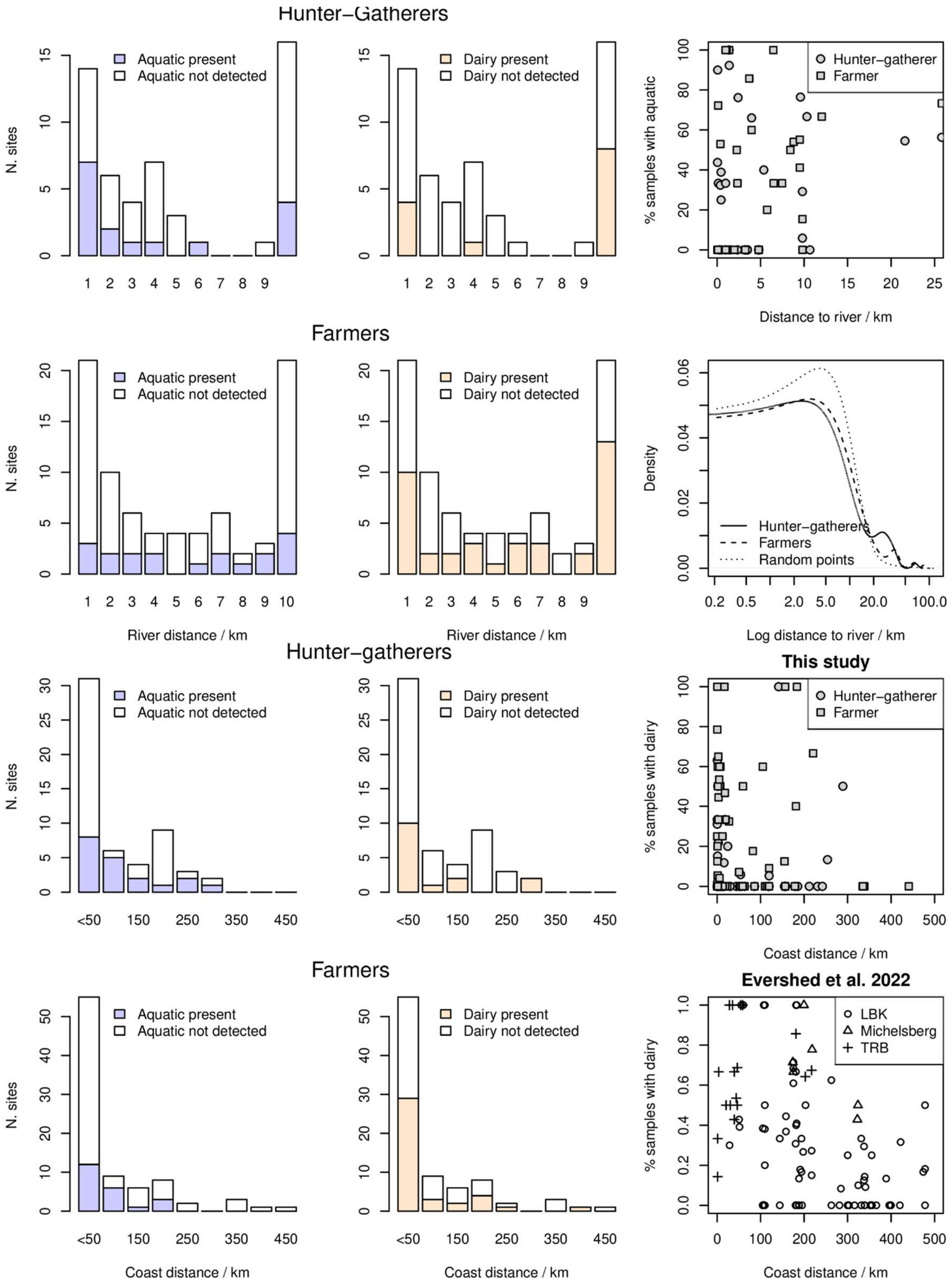

Fig. S3. Barplots showing frequency of sites with and without aquatic and dairy biomarkers, scatter plots of the prevalence of aquatic biomarkers versus river distance, the density distribution of sites with respect to distance from rivers, and scatter plots showing the prevalence of dairy biomarkers versus coastal distance, including comparison with the Evershed et al. (3) dataset.

Table S1. Summary table of new or previously published organic residues analysis by site.

Site number	Site	Region	Lat.	Long.	Sample number	HFG	Farmer
1	Abora I	D	56.92	26.87	33	15	18
2	Agernæs	B	55.62	10.29	1	1	0
3	Akali	D	58.40	27.20	13	8	5
4	Åkonge	B	55.58	11.55	36	12	24
5	Alksnynė 3	C	55.60	21.10	12	0	12
6	Aurebettjødno XVII	B	59.60	5.20	18	0	18
7	Bazel Sluis	A	51.14	4.32	8	0	8
8	Bebensee LA 76	B	53.87	10.30	4	0	4
9	Brandwijk	A	51.89	4.81	24	0	24
10	Celmi	D	57.04	25.94	1	0	1
11	Dąbki, site 9	B	54.38	16.33	174	129	45
12	Dąbki, Site 9	B	54.38	16.33	9	3	6
13	Daktariškė 5	C	55.80	22.39	73	18	55
14	Dakudava 5	C	53.79	25.54	1	0	1
15	De Bruin	A	51.83	4.80	20	20	0
16	Doel ‘Deurganckdok’	A	51.27	4.21	14	0	14
17	Drazdy 12	C	53.49	26.66	1	0	1
18	Dubičiai 2	C	54.00	24.70	2	0	2
19	Espoo Näkinkylä	D	60.20	24.60	2	0	2
20	FHM 1592 Ringkloster	B	56.01	9.97	20	20	0
21	FHM 1734 Frederiks Odde	B	56.03	10.26	1	1	0
22	FHM 1734 Norsminde	B	56.00	10.30	1	0	1
23	FHM 2033 Tybrind Vig	B	55.39	9.82	45	45	0
24	FHM 2911 Bjørnsholm	B	56.87	9.21	10	0	10

Site number	Site	Region	Lat.	Long.	Sample number	HFG	Farmer
25	FHM 3251 Åle	B	56.87	9.21	1	1	0
26	FHM 3705 Ronæs Skov	B	55.44	9.81	5	5	0
27	FHM 4014 Havnø	B	56.71	10.17	10	6	4
28	FHM 5163 Flynderhage	B	56.01	10.22	1	1	0
29	FHM 5184 Hjarnø	B	55.80	10.10	4	4	0
30	Flintbek LA 3	B	54.26	10.10	10	0	10
31	Gaigalinė 1	C	55.80	22.40	1	0	1
32	Gaigalinė 2	C	55.80	22.40	2	0	2
33	Gamborg Fjord	B	55.45	9.78	1	1	0
34	Garnys	C	55.13	25.97	16	16	0
35	Gribaša 4	C	54.04	24.70	2	0	2
36	Grube-Rosenhof LA 58	B	54.25	11.05	106	91	15
37	Hämeenlinna Hauho Perkiö	D	61.17	24.56	1	0	1
38	Hazendonk	A	51.86	4.83	9	0	9
39	Helsinki Malminkartano	D	60.25	24.86	1	0	1
40	Hüde I	A	53.11	8.47	58	0	58
41	Iru	D	59.46	24.90	2	0	2
42	Jägala Jõesuu V	D	59.47	25.17	4	4	0
43	Järvensuo 1	D	60.91	23.30	3	3	0
44	Järvensuo 2	D	60.91	23.30	3	0	3
45	Jordløse Mose VIII	B	55.20	10.16	1	0	1
46	Jordløse Mose XV	B	55.20	10.16	1	0	1
47	Jordløse Mose XX	B	55.20	10.16	9	0	9
48	Jordløse Mose XXI	B	55.20	10.16	1	0	1

Site number	Site	Region	Lat.	Long.	Sample number	HFG	Farmer
49	Kääpa	D	57.90	27.10	16	7	9
50	Kabeliai 23	C	53.95	24.29	1	1	0
51	Kaltanėnai	C	55.25	25.99	10	10	0
52	Karaviškės 6	C	54.03	24.68	5	0	5
53	Katros ištakos 1	C	54.00	24.70	2	0	2
54	Kayhude LA 8	B	53.80	10.10	4	4	0
55	Kesemölla	B	56.10	14.40	6	6	0
56	Kirkkonummi Backisåker I (Kvarnåker)	D	60.10	24.40	2	0	2
57	Kirkkonummi Koivistosveden	D	60.10	24.40	1	0	1
58	Kirkkonummi Tengå Nyåker	D	60.10	24.40	9	0	9
59	Kokemäki Kraviojankangas	D	61.30	22.40	13	13	0
60	Kunigiškiai	C	55.10	24.60	1	0	1
61	Kvietiniai	C	55.70	21.40	8	0	8
62	Lapinjärvi Malmbacken Norrby	D	60.62	26.20	3	0	3
63	Lemmetsa I	D	58.42	24.32	1	0	1
64	Löddesborg	B	55.73	12.97	24	24	0
65	Lommi (III)	D	59.40	28.32	27	27	0
66	Maglelyng 2	B	55.58	11.58	4	0	4
67	Maglelyng 3	B	55.58	11.58	1	0	1
68	Målevgård Mose	B	55.76	12.33	2	0	2
69	Margiai 1	C	54.00	24.70	5	1	4
70	Nakkila (Kiukainen) Uotinmäki	D	61.40	22.00	16	0	16
71	Narva Joaorg	D	59.40	28.20	51	34	17
72	Narva Jõesuu IIB	D	59.45	28.07	1	0	1

Site number	Site	Region	Lat.	Long.	Sample number	HFG	Farmer
73	Neravai	C	54.03	24.02	1	0	1
74	Neustadt LA 156	B	54.10	10.80	71	35	36
75	Neverkær Mose	B	55.40	10.09	1	0	1
76	Nida 1	C	55.30	21.00	79	3	76
77	Øgårde 3 (kar S)	B	55.59	11.54	1	0	1
78	Øgårde 5 (kar A)	B	55.59	11.54	1	0	1
79	Oldenburg-Dannau LA 77	B	54.30	10.85	18	0	18
80	Oudenaarde ‘Donk’	A	50.84	3.59	2	0	2
81	Papiškės 4	C	54.50	25.10	1	1	0
82	Polderweg	A	51.83	4.82	12	12	0
83	Pomarkku Myllytörmä/Patakoski	D	61.70	22.00	10	10	0
84	Porvoo Böle Munkby	D	60.44	25.77	2	0	2
85	Raasepori Telegrafberget	D	60.00	23.70	3	3	0
86	Riņņukalns	D	57.79	25.15	22	22	0
87	Roskilde Fjord III (300 m NNE of Kølholm)	B	55.81	12.06	1	0	1
88	Rzucewo 1	C	54.70	18.47	14	0	14
89	Šakės	C	54.10	24.60	1	1	0
90	Salpetermosen	B	55.90	12.30	1	0	1
91	Saltvik Härdalen 21.11	D	60.28	20.06	1	0	1
92	Šarnelė	C	56.10	22.00	4	0	4
93	Satrup-Förstermoor LA 71	B	54.69	9.63	1	1	0
94	Schlamersdorf LA 5	B	54.05	10.42	4	4	0
95	Seedorf LA 245	B	53.33	9.24	3	0	3

Site number	Site	Region	Lat.	Long.	Sample number	HFG	Farmer
96	Skirmantinė 1	C	55.80	22.40	1	0	1
97	Skogsmossen	B	59.60	16.50	9	0	9
98	Soldattorpet	B	59.30	18.10	21	21	0
99	Stenø	B	55.80	12.10	27	10	17
100	Šventoji 1	C	56.02	21.09	32	0	32
101	Šventoji 2	C	56.00	21.10	5	0	5
102	Šventoji 26	C	56.00	21.10	2	2	0
103	Šventoji 3	C	56.00	21.10	6	6	0
104	Šventoji 4	C	56.01	21.08	23	7	16
105	Šventoji 43	C	55.98	21.09	10	10	0
106	Šventoji 6	C	56.00	21.10	4	1	3
107	Swifterbant S2	A	52.58	5.58	17	0	17
108	Swifterbant S3	A	52.57	5.58	24	0	24
109	Swifterbant S4	A	52.57	5.58	38	0	38
110	Syltholm II (MLF00906-I)	B	54.65	11.39	40	11	29
111	Syltholm II (MLF00906-II)	B	54.65	11.39	35	14	21
112	Syltholm XIII (MLF00939-I)	B	54.60	11.30	28	7	21
113	Tallinn Müller's Field (Pärnu Rd. 37, 41)	D	59.43	24.74	6	0	6
114	Tamula I	D	57.84	26.99	20	6	14
115	Timmendorf-Nordmole	B	53.99	11.37	2	2	0
116	Tingbjerggård T1	B	55.59	11.58	1	0	1
117	Turku Jäkärlä	D	60.50	22.40	9	5	4
118	Turku Kotirinne	D	60.45	22.27	24	0	24
119	Ulkestrup Lyng	B	55.59	11.50	1	0	1

Site number	Site	Region	Lat.	Long.	Sample number	HFG	Farmer
120	Vantaa Jönsas-pohjoinen/itä Kaarela	D	60.26	24.86	1	0	1
121	Vantaa Maarinkunnas	D	60.30	25.10	1	1	0
122	Vantaa Stenkulla	D	60.30	25.10	7	7	0
123	Vasa	D	59.42	28.14	5	0	5
124	Västanfjärd Galtarby II	D	60.05	22.67	2	0	2
125	Vik	B	55.60	14.30	1	1	0
126	Virolahti Meskäärtty	D	60.52	27.49	11	11	0
127	Wangels LA 505	B	54.27	10.77	20	0	20
128	Wangels LA 69	B	54.28	10.78	15	0	15
129	Žemaitiškė 1	C	55.30	26.10	4	0	4
130	Žemaitiškė 2	C	55.30	26.10	2	2	0
131	Žemaitiškė 3	C	55.30	26.10	2	1	1

Dataset S1 (separate file). Molecular and isotopic measurements of lipids extracted from circum-baltic vessels.

Dataset S2 (separate file). Frequency of dairy fat residues across sites from Northern Europe.

References

1. S. N. Wood, Thin plate regression splines. *J. R. Stat. Soc. Series B Stat. Methodol.* **65**, 95–114 (2003).

2. M. Cubas, *et al.*, Latitudinal gradient in dairy production with the introduction of farming in Atlantic Europe. *Nat. Commun.* **11**, 2036 (2020).

3. R. P. Evershed, *et al.*, Dairying, diseases and the evolution of lactase persistence in Europe. *Nature*, 1–10 (2022).

4. B. Courel, *et al.*, The use of early pottery by hunter-gatherers of the Eastern European forest-steppe. *Quat. Sci. Rev.* **269**, 107143 (2021).

5. K. J. Gron, *et al.*, Nitrogen isotope evidence for manuring of early Neolithic Funnel Beaker Culture cereals from Stensborg, Sweden. *Journal of Archaeological Science: Reports* **14**, 575–579 (2017).

6. K. J. Gron, *et al.*, Archaeological cereals as an isotope record of long-term soil health and anthropogenic amendment in southern Scandinavia. *Quat. Sci. Rev.* **253**, 106762 (2021).

7. D. Filipović, *et al.*, Middle-Neolithic agricultural practices in the Oldenburger Graben wetlands, northern Germany: First results of the analysis of arable weeds and stable isotopes. *Holocene* **29**, 1587–1595 (2019).

8. A. Mueller-Bieniek, *et al.*, Spatial and temporal patterns in Neolithic and Bronze Age agriculture in Poland based on the stable carbon and nitrogen isotopic composition of cereal grains. *Journal of Archaeological Science: Reports* **27** (2019).

9. A. Bogaard, *et al.*, Crop manuring and intensive land management by Europe's first farmers. *Proc. Natl. Acad. Sci. U. S. A.* **110**, 12589–12594 (2013).
